# The role of the oviduct and extracellular vesicles during early embryo development in bovine

**DOI:** 10.1590/1984-3143-AR2022-0015

**Published:** 2022-04-20

**Authors:** Natália Marins Bastos, Juliana Germano Ferst, Rodrigo Silva Goulart, Juliano Coelho da Silveira

**Affiliations:** 1 Departamento de Medicina Veterinária, Faculdade de Zootecnia e Engenharia de Alimentos, Universidade de São Paulo, Pirassununga, SP, Brasil; 2 Departamento de Zootecnia, Faculdade de Zootecnia e Engenharia de Alimentos, Universidade de São Paulo, Pirassununga, SP, Brasil

**Keywords:** oviductal environment, oviductal fluid, cell communication

## Abstract

The oviduct is an important reproductive structure that connects the ovary to the uterus and takes place to important events such as oocyte final maturation, fertilization and early embryonic development. Thus, gametes and embryo can be directly influenced by the oviductal microenvironment composed by epithelial cells such secretory and ciliated cells and oviductal fluid. The oviduct composition is anatomically dynamic and is under ovarian hormones control. The oviductal fluid provides protection, nourishment and transport to gametes and embryo and allows interaction to oviductal epithelial cells. All these functions together allows the oviduct to provides the ideal environment to the early reproductive events. Extracellular vesicles (EVs) are biological nanoparticles that mediates cell communication and are present at oviductal fluid and plays an important role in gametes/embryo - oviductal cells communication. This review will present the ability of the oviducts based on its dynamic and systemic changes during reproductive events, as well as the contribution of EVs in this process.

## Introduction

*In vitro* embryo production (IVEP) is a very well-established reproductive biotechnology, used worldwide and capable to contribute to the increasing genetic merit and consequently improvement of different domestic species. However, the use of IVEP exposes gametes and embryos to non-physiological spatial and temporal conditions (Besenfelder et al., 2020). Furthermore, *in vivo* derived embryos still have superior quality and developmental potential than those produced *in vitro* (Rizos et al., 2002a, b). Thus, the IVEP is efficient in producing embryos, but it still does not completely mimic the physiological ovarian and oviductal environments. During the short period of early embryonic development within the oviduct, the embryo will activate its genome, thus comprising a sensitive and important period that can determine the subsequent stages of its development.

In the *in vivo* situation, the oviduct takes place to important events for the reproductive function, such as final oocyte maturation, fertilization and early embryonic development. The oviduct can provide a favorable and dynamic microenvironment for ideal functioning and development of these processes. Interestingly the oviduct of different species has similar biological properties; however, the time that the embryo resides and undergoes modifications is not the same. Bovine embryos develop within the oviduct within 4 days after ovulation (Kölle et al., 2009), 4 days in human (Aplin, 2003), 5.5 days in equine (Freeman et al., 1991) and 4 days in mice (Potts and Wilson, 1967), demonstrating the importance for studying the events that take place in the oviduct.

The oviduct fluid that bathes gametes and embryos contain substrates and co-factors that help to create the oviductal environment prepared to the embryo cleavage and development. Furthermore, present in the oviduct fluid, extracellular vesicles (EVs) are nanoparticles that mediate cell communication acting as vectors of biological information. In the oviduct, the EVs plays an important role once they interact with gametes-embryo and oviductal epithelial cells acting in this bidirectional communication. Thus, oviductal EVs can modulates the oviductal environment and influences the reproductive events that takes place there, and also the oviduct-embryo interaction in order to contribute with maternal-embryonic communication even before the recognition of pregnancy (Mazzarella et al., 2021). Therefore, this review will present the adaptability of the oviduct during reproductive events based on its dynamic and systemic changes, as well as the contribution of EVs in this process.

## Composition and importance of the oviduct environment for early embryonic development

The oviduct is a small, elongated and tubular structure that connects the ovary to the uterus and is formed by a fibromuscular complex composed of layers such as mucosa, muscle and connective serosa (Besenfelder et al., 2012, 2020; Avilés et al., 2015). These layers’ structure and composition depends on the three different anatomical portions that make up the oviduct: infundibulum, ampulla and isthmus (Besenfelder et al., 2012, 2020; Avilés et al., 2015). The infundibulum is responsible for capturing recently ovulated cumulus-oocyte-complexes (COCs) (Besenfelder et al., 2012; Avilés et al., 2015). The cumulus cells extracellular matrix filaments are able to adhere to the infundibulum cells glycocalyx and enter the oviduct at the ampulla region where the oocyte maturation process is completed and fertilization takes place (Kölle et al., 2009; Li and Winuthayanon, 2017). The cilia beating is responsible for creating a negative pressure and microtubule movement, which will produce a current flow that helps the COC movement throughout the oviduct towards the uterine (Olsen et al., 2018). Bad quality oocytes move faster through the oviduct by floating in oviductal lumen, demonstrating that there is function related to the movement as well as the capability of COC and oviduct to recognize themselves (Kölle et al., 2009). Female and male gametes enter at oviduct from opposite sides but oocyte and sperm meet in the ampulla (Besenfelder et al., 2020). In order to the sperm arrive at the oviduct, uterine contractibility as well as oviduct secretions play an important role directing the sperm towards the ampulla (Hawk, 1983; Suarez, 2008). Then, on isthmic region crucial processes related to pre-implantation embryo development takes place. During the time that the embryo stays at isthmus, besides the initial development, the major gene activation happens starting at the 8-cell stage (Memili and First, 2000) suggesting that this oviductal region has an important role through the subsequently embryo development at uterus.

A size comparison between the different parts of the oviduct demonstrated that the ampullary lumen is large and filled with primary and secondary folds, while the isthmic lumen is smaller and constituted only with primary folds (Besenfelder et al., 2020). The mucosa is constituted by epithelial cells that can be ciliated or secretory cells, and these cells proportion depends on the oviducts anatomical portion and the ovarian cycle stage. Endocrine mechanisms, mainly controlled by steroid hormones (estrogen and progesterone) are well known to mediate morphological, physiological and molecular changes in the oviduct (Gonella-Diaza et al., 2017; Almiñana et al., 2018; Gonella-Diaza et al., 2018). In response to the high concentrations of preovulatory estrogen (E2), the oviduct initiates morphogenic and proliferative processes in the ampulla lumen, increasing the number of secretory cells and the functional area of the epithelium (Gonella-Diaza et al., 2017). Thus, the ampulla prepares to become receptive to the COC and sperm cells. After fertilization, the number of secretory cells continuously decreases and, during the embryo first cleavages, the isthmus is mostly composed by ciliated cells (Kölle et al., 2009). COCs and embryos are immobile and must be transported through the oviductal anatomical structures by a combination of factors: waves of smooth muscle layer contraction and relaxation (longitudinal and circular), ciliary beating of epithelial cells and follicular fluid flow. These mechanisms are mostly controlled by steroid hormones and prostaglandins (Amini et al., 2015). Apparently, in a pre-ovulatory stage, E2 modulates the action of prostaglandins (PGE2 and PGF2α) in the oviduct (Lindblom et al., 1980) and is related to muscle contraction, increased frequency of ciliary beat and increased oviductal fluid volume, as it increases the number of secretory cells (Valle et al., 2007; Huang et al., 2015; Gonella-Diaza et al., 2017). Progesterone (P4) seems to have the opposite effect to E2 (Lindblom et al., 1980), once this hormone acts in the muscle relaxation and decreased frequency of ciliary beat.

The sperm, even having their own movement, must undergo morphophysiological changes to reach fertilizing capacity such as hyperactivation and acrosome reaction. In cows, the semen is ejaculated into vagina, against the cervix, where the natural selection occurs by the cervical mucus flow and only the motile sperm can advance towards the uterus (Coy et al., 2012; Li and Winuthayanon, 2017). Besides their own motility, the sperm moves through the uterus due to muscular contractions, ciliary beat and fluid flow that helps the healthy sperm to arrive at uterotubal junction (Hawk, 1983). Once in the oviduct, sperm interact with isthmic epithelial cells. The oviduct guides the sperm to the fertilization site and helps in this process, but first provides the formation of a sperm reservoir and enabling the activation of Ca^2+^ influx in the sperm in order to initiate the flagellum hyperactivation process (Miki and Clapham, 2013). Once this occurs, the sperm is able to swim against the oviduct flow to find the COC to be fertilized (Kölle et al., 2009). The follicular fluid present at the ampulla after ovulation contains progesterone (Saint-Dizier et al., 2020) and chemoattractants that helps to bring the sperm closer to the COC within the ampullary region. Furthermore, proteins and phospholipids present in the ampulla lumen can influence the sperm fertilizing capacity inducing the acrosome reaction (Griffiths et al., 2008). Thus, the oviduct and its fluid are able to guide the sperm and provide subsidies for it to become fertile in addition to sense the presence of the sperm cells and adjust proteins and antioxidants concentration possibly reducing sperm stress. Also, the increase in E2 concentration induces the production of GPX4 (Glutathione peroxidation 4) in the oviduct, indicating an antioxidant defense mechanism for gametes and future embryonic development (Lapointe et al., 2005).

Gametic and embryo transport play an important role, since to be fertilized the COC and sperm must be at the proper time and place for fertilization, and the early development embryo must to exit from the oviduct to carry on development otherwise it can implant in the wrong place or generate an ectopic pregnancy. Furthermore, embryonic development and transport are simultaneous events under physiological conditions (Li and Winuthayanon, 2017). Embryonic movement, acts to prevent the accumulation of harmful by-products to embryonic development once allows full exposure of the embryo to the medium providing the appropriate access to nutrients and preventing metabolic stress during the first cleavages (Hu and Yu, 2017).

In addition, a recent study demonstrated that the embryo presence alters the miRNA profile of the isthmic cells generating an inflammatory type response (Mazzarella et al., 2021), suggesting that the embryo presence modulates the oviductal epithelial cells. In goats, the nutritional plan influences the ampullary epithelial cells protein profile (Fernandes et al., 2018). Furthermore, environmental factors such as the animal's energy balance also appear to have an influence on the oviduct dynamics. Therefore, the oviduct is a dynamic and adaptable structure able to respond to situations in which it is exposed and is not just a simple organ for transporting COCs and embryos.

## Composition and importance of oviductal fluid

Until the establishment intimate contact between mother and embryo, embryonic development is directly influenced by secreted products by oviductal and endometrial epithelial cells (Binelli et al., 2018). Oviductal fluid, synthesized primarily by secretory epithelial cells, is also composed of transudate from the systemic circulation and supplemented by follicular fluid upon ovulation (Li and Winuthayanon, 2017; Olsen et al., 2018; Besenfelder et al., 2020). Prior to fertilization, oviductal fluid is responsible for protecting and guiding sperm and COCs (Li and Winuthayanon, 2017). After fertilization, the oviductal fluid role is responsible to nourish, protect and assist the transport of the pre-implantation embryo (Olsen et al., 2018) as well as to provide optimal pH and stable temperature (Li and Winuthayanon, 2017). Its composition is anatomically dynamic and influenced by early developing embryo presence (Rodríguez-Alonso et al., 2020a; Mazzarella et al., 2021). Interestingly, the embryo presence is capable of modulating the oviduct environment, being able to acquire components of the maternal environment.

The volume of oviductal fluid is dependent on the estrous cycle and varies among species, but in general, in mammals, the highest production is during the end of estrus and beginning of diestrus (Leese et al., 2001), when the oviduct prepares to receive gametes and embryo by increasing the number of secretory epithelial cells as well as lumen size (Leese et al., 2001; Gonella-Diaza et al., 2017). In addition, the increase in oviductal fluid volume during this period is also due to the follicular fluid coming from the recently ovulated follicle (Saint-Dizier et al., 2020). Thus, the estimated volume of the oviductal fluid in bovine is 1-3 mL per day on day one of the estrus cycle, and 0.1-0.2 mL per day at luteal phase (Kavanaugh and Killian, 1988).

Oviductal fluid is composed by substrates and cofactors linked to oocyte maturation, oocyte fertilization and early embryo development such as glucose, arginine, serum albumin, transferrin, glycoprotein, galactose, immunoglobulins, lactate, pyruvate, bicarbonate, cytokines, growth factors, amino acids, enzymes, hormones and EVs (Beier, 1974; Binelli et al., 2018; Rodríguez-Alonso et al., 2020b; Saint-Dizier et al., 2020). These constituents’ concentration varies among species, estrous cycle stage and oviduct anatomical portion (Hu and Yu, 2017; Rodríguez-Alonso et al., 2020a). This is probably due to the different metabolic needs of gametes and embryo as they pass through oviduct.

Oocytes, sperm and embryos in early developmental stage, use oxidative metabolism to obtain energy. During the first cleavages, until the morula stage, embryonic mitochondria are not yet mature, so simple sugars such as pyruvate and lactate (Rodríguez-Alonso et al., 2020a) and high oxygen concentration (Hu and Yu, 2017) are essential for oxidative phosphorylation that provides energy for the first cleavages. Pyruvate is an important sugar for oxidative phosphorylation, but is mostly intended for lactate conversion. Lactate is a simple sugar that is essential for early embryonic development, which acts to protect cells at the cleavage stage from toxins, oxidative stress and infections (Hu and Yu, 2017). In cyclic and pregnant cows, the ampullary lactate concentration is higher than in the isthmus (Rodríguez-Alonso et al., 2020a). This suggests that besides oviduct adaptation to the estrous cycle and embryo presence, oviduct is able to produce lactate to help with embryo metabolism. The ATP production increases even before first cleavage, but the early embryo metabolism has low metabolic and nutrient uptake due to the minimal cell growth and membrane biosynthesis (Li and Winuthayanon, 2017; Hu and Yu, 2017). The early developing embryo has a low metabolic requirement originating the “quiet embryo” hypothesis (Leese, 2002). Leese (2002) suggests that the embryo should remain “quiet”, with reduced metabolism during early development in order to minimize the production of reactive oxygen species and other metabolic products harmful to the embryo during this vulnerable time period. Thus, is important that the oviduct can detect the embryo presence in order to regulate the right amounts of molecules to be secreted in its lumen.

As the embryo develops, mitochondrial maturation occurs and the embryo metabolism, which was previously oxidative, becomes glycolytic. Thus, the presence of glucose becomes essential for the compaction and embryonic genome activation (Li and Winuthayanon, 2017; Hu and Yu, 2017). The absence of glucose during compaction irreversibly decreases cell proliferation and increases apoptosis and oxidative stress (Jansen et al., 2009; Pantaleon et al., 2007). The concentration of glucose in the oviductal fluid is not static, that is, it varies according to oviductal need; however, during the stage of embryonic oxidative metabolism, it is essential that the concentration of this sugar stay low to avoid metabolic stress (Hu and Yu, 2017). Changes in glucose availability can accelerate or delay key events during the major genome activation shifting from oxidative to glycolytic metabolism (Harvey, 2019).

Therefore, as the gametes and embryo transit through the oviduct, the need for nutrients available in the oviductal fluid varies according to the gametic-embryonic metabolism. The oviductal fluid composition contributes to the embryo development and could modulate the maternal-embryonic communication processes even before the maternal gestation recognition. Present in oviductal fluid, the EVs acts in this modulation by mediating the bidirectional communication between oviductal epithelial cells (mother) and gametic-embryonic cells.

## Role of EVs in oviductal dynamics and maternal-embryonic communication

In addition to substrates and cofactors, EVs are also present in the oviduct fluid (Al-Dossary et al., 2013) and play important roles in the oviduct dynamics environment and maternal-embryonic communication (Mazzarella et al., 2021). EVs are evolutionarily conserved mediators of cell communication (Fu et al., 2020). These biological nanoparticles use extracellular fluids to diffuse and interact with target cells in order to transfer their contents (Silveira et al., 2015, 2018; Gross et al., 2017) acting as vectors of biological information and being able to modify the cell function of recipient organs (Lawson et al., 2017). EVs are nanoparticles secreted by different cell types and initially classified into exosomes and microvesicles. Exosomes are small EVs originated from endosomes and have a 30-150 nm diameter; microvesicles are larger EVs, having 100-1000 nm and originated in the plasma membrane (Machtinger et al., 2016; van Niel et al., 2018). The EVs isolated by serial centrifugation, filtered in > 0.20 µm filter and validated nanoparticle tracking analysis (*Nanotracking*), transmitted electron microscopy and specific proteins by western blot, are recently classified as small EVs, according to Minimal Information for Studies of Extracellular Vesicles guidelines (Théry et al., 2018). Molecules present on the surface of EVs allows the interaction with target cells through their adhesion to lipids and receptor ligands, allowing their entry into cells through endocytosis or pinocytosis (Machtinger et al., 2016; Gross et al., 2017). In reproduction it was already demonstrated that EVs are able to modulate, cumulus-oocyte-complex maturation, embryonic development (Battaglia et al., 2019), as well as changes in global DNA methylation and hydroxymethylation levels of bovine embryos (Silveira et al., 2017).

**Figure 1:**
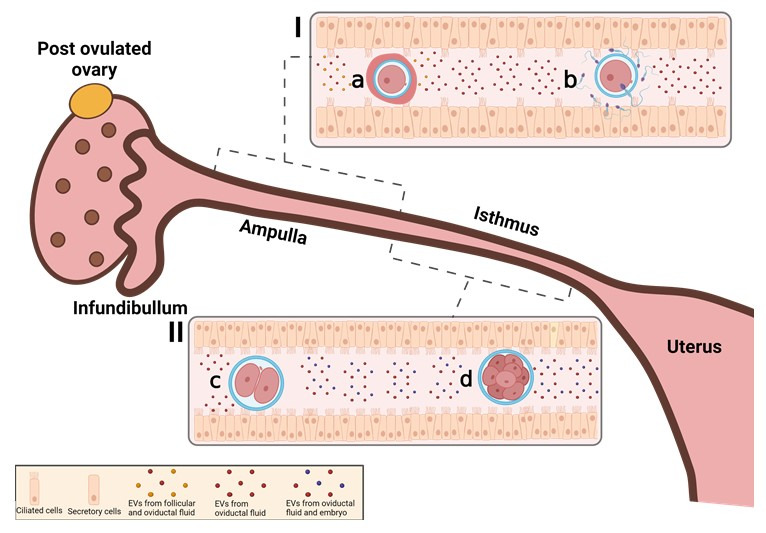
Reproductive events occurring in the oviduct mediated by extracellular vesicles. Schematic representation of female reproductive tract in post ovulation stage of estrus cycle and oviductal anatomic regions. The infundibulum is responsible for capturing recently ovulated cumulus oocyte complexes (COC). **(I)**The ampullary region is mainly composed by secretory epithelial cells which allows a high production of oviductal fluid which is also composed by follicular fluid. **(a)** Together these vesicles act at the oocyte final maturation; **(b)** At the ampulla region cumulus oocyte complexes and sperm will meet, and subsequently initiates the fertilization process. **(II)** The isthmic region is mainly composed by ciliated cells; **(c)** Once the fertilization happened, the initial embryo development occurs; **(d)** The embryo develops, activates its genome and modulates the isthmic region mediated by embryo and oviductal extracellular vesicles. This **figure was** Created with BioRender.com.

In the oviduct, EVs were identified as key mediators components in the interaction between gametes and embryo, contributing to the pregnancy success (Almiñana and Bauersachs, 2020). The EVs that make up the oviduct fluid can have different origins: i) follicular fluid upon ovulation; ii) secreted by oviduct epithelial cells; iii) secreted by gametes; iv) secreted by the embryo (Figure 1). Although it is difficult to distinguish these EVs origins, in general they act on physiological and molecular functions influencing oocyte maturation, spermatic hyperactivation and embryonic development (Harris et al., 2020). Importantly, it is possible that EVs act as fine-tuners of early reproductive events since these events can occur out of the reproductive tract. These interactions are possible due to the fact that EVs contain bioactive material such as proteins, lipids, mRNAs and miRNAs that are transferred to the target cells (Valadi et al., 2007; Silveira et al., 2015; Al-Dossary and Martin-DeLeon, 2016). Thus, the content of EVs in the oviduct can modulate cell function by increasing the delivery of transcripts, miRNAs and proteins, thus affecting translation of mRNAs into functional proteins (Bauersachs and Almiñana, 2020). MiRNAs are stable small non-coding RNA molecules involved in several cellular processes and indispensable for animal development, cell differentiation and homeostasis (Bartel, 2009, 2018; Gebert and Macrae, 2019). The role of miRNAs in the reproductive cycle is essential for gamete development, oocyte maturation, fertilization and early embryonic development (Hayashi et al., 2008). In the extracellular environment, miRNAs are fragile and, once inside the EVs, they are protected from degradation and can act as information vectors (Fu et al., 2020). Furthermore, EVs have powerful systemic access to the most varied and distant cells enabling miRNAs to fulfill autocrine, paracrine and endocrine signaling functions (Gross et al., 2017). Thus, EVs carrying messages including miRNAs could play an important role within the oviduct once these messages are different depending on ovarian cycle stage or embryos presence/absence (Table 1).

**Table 1:** The differential expression of miRNA content of oviductal extracellular vesicles and their predictive biological associated pathways.

**Ovarian cycle stage** **1**	**Embryo presence**	**Oviductal region** **2**	**miRNAs**	**Biological associated pathways**	**Reference**
S4 compared to other stages	No	All	miR-1291, miR-323, miR-631, miR130a, miR-433, miR-489, miR382, miR378	GnRH signaling pathway, FoxO signaling pathway, Vascular smooth muscle contraction, Signaling pathways puripotency of stem cells, Wnt signaling pathway	Hamdi et al. (2021)
S2	Yes	Isthmus	miR-126-5p, miR129, miR-140, miR-188, miR-219, miR345-3p, miR-4523, miR-760-3p	Metabolic pathways, PI3K-Akt signaling pathway, MAPK signaling pathway, Endocytosis, Ras signaling pathway	Mazzarella et al. (2021)
	No		miR-331-5p	cAMP signaling pathway, Insulin signaling pathway, Regulation of actin cytoskeleton, Wnt signaling pathway, Focal adhesion	
S1 and S4	No	All	miR-10b-5p	Pyrimidine metabolism	Almiñana et al. (2018)
			miR-423-5p	Fatty acid biosynthesis, Fatty acid metabolism	
			miR-449a	Carbon metabolism, HIF-1 signaling pathway	
			miR-375	Hippo signaling pathway, Amino sugar and nucleotide sugar metabolism	
			miR-24-3p	Fatty acid biosynthesis, Vitamin B6 metabolism, Endocytosis, Hippo signaling pathway, Bacterial invasion of epithelial cells	
			miR-148a-3p	Fatty acid biosynthesis, Steroid biosynthesis, Oocyte meiosis, Progesterone mediated oocyte maturation, FoxO signaling pathway	
			miR-429	Axon guidance, FC gamma R-mediated phagocytosis, Steroid biosynthesis, Progesterone mediated oocyte maturation, Gap junction	
			miR-34b-3p	Glycosaminoglycan degradation	
			miR-200b-3p	Ras signaling pathway, Neurotrophin signaling pathway	
			miR-92a-3p	Cell cycle, Adherens junction, Thyroid hormone signaling pathway, FoxO signaling pathway, RNA transport, Signaling pathways regulating pluripotency of stem cells	
			miR-151a-3p	Biosynthesis of unsaturated fatty acids, Fatty acid metabolism	
			miR-30d-5p	Mucin type O-, Glycan biosynthesis, Oocyte meiosis, Ubiquitin mediated proteolysis, mRNA surveillance pathway	
			miR-125b-5p	ErbB signaling pathway, Regulation of actin cytoskeleton	

^1^Ovarian cycle stage: S1: postovulatory-stage; S2: early luteal phase; S3: late luteal phase; S4: pre-ovulatory stage. ^2^Oviductal region: The total oviductal structure was flushed (ampullary and isthmic region).

In the oviduct, embryos stay and develop in a short period of time (4-5 days), but this can have great consequences at later stages of development (Fu et al., 2020). Zygotes and pre-implantation embryos are not in direct contact with the oviduct because they still have the zona pellucida (ZP), which is resistant to the uptake of exogenous genetic material, although the biological action of EVs allows these molecules to enter the ZP and perform functions in the embryo (Fu et al., 2020). Interestingly, the oviductal EVs miRNAs cargo are related to embryonic development, embryonic morphology and implantation (Almiñana et al., 2018). EVs originated from oviductal fluid and *in vitro* culture of bovine oviduct epithelial cell (BOEC) were able to internalize in *in vitro* produced bovine embryos, increasing production rates, prolonging embryo survival and to improving their quality and cryoprotection (Lopera-Vasquez et al., 2016; Almiñana et al., 2017). Additionally, oviductal EVs contain mRNAs associated with epigenetic DNA modifications, indicating that these biological nanoparticles can control chromatin modification and epigenetic regulation in the developing embryo (Almiñana et al., 2018). Moreover, besides the oviductal EVs supplementation in IVEP did not affect the blastocyst production rates and embryo cryotolerance, the oviductal EVs were able to modulate the blastocyst phospholipid content by making it more abundant in phosphatidylcholines (PC), phosphatidylethanolamines (PE) and sphingomyelins (SM) with long-chain fatty acids (Banliat et al., 2020). This is interestingly because when analyzing the oviductal EVs lipidic content, authors found that the overabundant lipids in blastocysts were 100% also present in oviductal EVs (Banliat et al., 2020). This suggests that in vitro produced embryos can incorporate the lipidic EVs content which may modulate the embryonic lipidic metabolism. Thus, suggesting that EVs from the oviduct can impact bovine embryos *in vivo* and *in vitro*.

As already discussed, the oviduct is a dynamic structure capable of adapting during reproductive events (maturation, fertilization and early embryonic development) under the action of ovarian hormones. According to Rodríguez-Alonso et al. (2020a) oviductal fluid composition is anatomically dynamic and affected by embryo presence. Since the content of EVs reflects the cells of origin, changes in the epithelium cellular morphology can induce changes in the biological functions of EVs (Al-Dossary and Martin-DeLeon, 2016; Almiñana and Bauersachs, 2020). These changes could lead to changes in secretion and contents of EVs during the estrous cycle. At the estrous cycle phases (postovulatory-stage, early luteal phase, late luteal phase and pre-ovulatory stage), the oviductal fluid EVs RNA and protein contents are different and many of these molecules are related to gametic interaction and pre-implantation embryo development (Almiñana et al., 2018). Another recent study assessing the miRNA profile from oviductal and uterine fluid EVs, showed that the estrous cycle change the EV cargo (Hamdi et al., 2021). Together, this information suggests that these EVs are under hormonal control and indicates the crucial role of EVs in reproductive events. Furthermore, the isthmic EVs culture medium supplementation used in IVEP induces greater blastocysts rates (91.3%) when compared to ampullary EVs (62.2%) (Lopera-Vasquez et al., 2016). In addition, the results suggest that isthmus EVs may contribute to the normal regulation of the methylation pattern in embryos and improvement of embryonic cryopreservation, indicating that the content of EVs may be anatomically variable. However, studies analyzing the EVs content through the oviductal regions should be performed to better elucidate their function at the distinct anatomical regions. Finally, in a recent study, although the size and concentration of EVs has not been altered, the miRNA profile from isthmus EVs of Nelore cows is altered in the presence of a single embryo (Mazzarella et al., 2021). Using a miRNAs analysis, the authors identified predicted biological pathways regulated by EVs miRNAs which were involved with the immune system suggesting that EVs can possibly mediate maternal-embryonic communication even before pregnancy recognition. Additionally, EVs from good quality embryo and degenerating embryo were supplemented to primary BOEC monolayer culture (Dissanayake et al., 2020). Genes related to interferon-τ-induced genes were upregulated at the BOEC monolayer suggesting that the embryos EVs modulates the oviduct in response to their quality (Dissanayake et al., 2020). Furthermore, it was already demonstrated that EVs can carry molecular signals in response to environmental factors such as environmental stress and body energy balance (Tesfaye et al., 2020).

Thus, EVs can act mediating the bidirectional crosstalk between mother and gametes/embryo, helping to fine tuning the oviduct and endometrium contributing to successful embryo development and implantation. However, these types of studies that analyze the role of these EVs in embryonic development are still recent (Al-Dossary et al., 2013) and compose promising lines of research, once they importance to oviductal environment and embryo development. Additionally, it is possible that EVs generate specific biological environments providing the physiological basis for oocyte maturation, fertilization and early embryonic development within the oviduct.

Despite the evidences that oviductal EVs and their contents can play important roles in oocyte maturation in the oviduct, due to the recent discovery of oviductal EVs (Al-Dossary et al., 2013) and the difficulty of obtaining oviductal samples, additional analysis are still necessary to demonstrate the action of these nanoparticles in the final oocyte maturation.

## Conclusions

Anatomically the oviduct is a small structure where the embryo develops in a short period of time (4-5 days); furthermore this reproductive structure has a huge importance at early embryonic development and subsequently pregnancy establishment. This is due to the unique microenvironment that the oviduct provides to gametes and embryos, built under the influence of ovarian hormones, oviduct fluid composition and EVs mediation. As future perspectives, the understanding of EVs contribution to the oviductal environment fine tuning can help to better mimic the *in vitro* environment during IVEP and contribute to increase quality of *in vitro* produced embryo.
